# Role of Betel Nut in Liver Toxicity in Oral Submucous Fibrosis and Oral Cancer Patients: A Case-Control Study

**DOI:** 10.7759/cureus.48562

**Published:** 2023-11-09

**Authors:** Shivani S Mittal, Swapnil Mohod, Vidya Lohe, Shraddha Patel, Monika Khubchandani, Monal M Kukde

**Affiliations:** 1 Department of Oral Medicine and Radiology, Sharad Pawar Dental College and Hospital, Datta Meghe Institute of Higher Education and Research (Deemed to be University), Wardha, IND; 2 Department of Pediatric Dentistry, Sharad Pawar Dental College and Hospital, Datta Meghe Institute of Higher Education and Research (Deemed to be University), Wardha, IND; 3 Department of Dentistry, Datta Meghe Medical College and Shalinitai Meghe Hospital, Nagpur, IND

**Keywords:** oral submucous fibrosis, liver function test, oral cancer, liver toxicity, betel nut

## Abstract

Background: In 2004, the International Agency for Research on Cancer (IARC) revised its conclusion that betel quid, both with and without tobacco, as well as areca nut alone, was carcinogenic to humans. Areca nut may enhance chemical hepatocarcinogenesis. Researchers have studied the role of areca nut components in the etiology of oral submucous fibrosis (OSF) for the past two decades.

Objectives: In this, we will study the role of betel nut chewing on the liver and its correlation with the occurrence of OSF and oral cancer.

Methodology: It is a type of case-control study for a duration of three months. A total of 60 subjects were selected based on the selected groups and exclusion criteria. A detailed case history was taken, and after that blood samples were collected for conducting liver function tests. After the collection of reports from the labs, the results were assessed, analyzed, and correlated with the case history of each subject.

Results: This research aids in the identification of a link between the occurrence of OSF, oral squamous cell carcinoma (OSCC) liver damage, and the practice of eating betel nuts. Chewing betel quid on a regular basis appears to be a separate risk factor for liver damage, OSCC, and OSF.

Conclusion: This assessment of liver function with case history in each subject aids in providing an improvised and prioritized method for the early diagnosis of liver misfunctioning in the patient with OSF or Oral Cancer due to a common etiological factor, that is betel nut.

## Introduction

The primary factor for heightened incidence and mortality rate is the high prevalence of betel quid (BQ) chewing, which affects more than one-fourth of the Indian population [1]. The fruit of the Areca catechu tree produces areca nuts, which are known for their alkaloids (especially arecoline) and tannins, which are the nut's most potent active components [2]. BQ ranks as the fourth most consumed drug; for its mild psychoactive and cholinergic effects [3]. It is believed that around 600 million people worldwide utilize betel nut for social traditions, religious practices, and cultural rites, or as a hallucinogenic drug [4]. There were no significant associations between betel nut consumption and age, sex, salary, education, or marital status [5]. Chewing betel nut (BQ) is a common oral practice that may have a connection to the development of oral cancer. The IARC came to the conclusion that chewing tobacco-laced BQ was carcinogenic to humans in 1987 [6]. In 2004, the IARC updated their assessment that BQ with and without tobacco, as well as areca nut alone, were carcinogenic to humans [2].

Nonalcoholic fatty liver disease (NAFLD) is one of the most frequent liver illnesses nowadays. It has been linked to obesity, hypertension, dyslipidemia, type 2 diabetes, cardiovascular disease, and even death. Furthermore, NAFLD raises the risk of liver fibrosis, cirrhosis, and hepatocellular cancer [4]. Hepatocellular carcinoma is one of the most frequent, dangerous, and common malignant human tumors. Chewing betel nut which contain nitrosamines, may cause methylation and cyanoethylation of liver DNA, causing hepatocyte genotoxicity and, in the long run, liver cancer. Chemically, areca nuts may cause hepatocarcinogenesis. The betel leaf, on the other hand, has significant quantities (15 m-1g fresh weight) of safrole, which is known to induce liver cancer in rats [7].

Oral submucous fibrosis (OSF), is a potentially malignant disorder predominantly seen in people of Asian descent [8]. It is a chronic inflammatory condition characterized by progressive fibrosis of oral connective tissues, which limits mouth opening and makes speaking, eating, and swallowing more difficult. It is proved to be an oral precancerous disease with a malignant transition rate of around 7.6% [9].

Several studies show overwhelming evidence that areca nut is the primary etiological cause for OSF. Over the last two decades, researchers have thoroughly investigated the function of areca nut components in the pathogenesis of OSF. It is clear that fibrosis and hyalinization of subepithelial tissues account for the majority of clinical characteristics seen in explaining the etiology and pathophysiology of this illness [8].

Consumption of betel nuts is a significant oral cancer risk factor [10]. The amount of quid ingested daily and the duration of chewing have a dose-response relationship with the risk of oral cancer [11].

Therefore, this study aids in the early detection of liver toxicity in patients with OSF and oral cancer, if any; it may also demonstrate that OSF and liver toxicity can co-occur with betel nut as an independent risk factor; and it may aid in the reduction of the incidence of a severe condition in patients with liver toxicity or cancer.

## Materials and methods

After gaining approval from the Institutional Ethics Committee in its meeting held on June 23, 2022; ref. no. DMIMS(DU)/IEC/2022/1170. This research was carried out at the Sharad Pawar Dental College, Datta Meghe Institute of Medical Sciences, Wardha, in the Department of Oral Medicine and Radiology.

A total of 60 subjects who belonged to the central India region were selected; 15 in each group based on the following groups, inclusion and exclusion criteria. Group 1 - patient without betel nut chewing habit with no history of OSF and oral squamous cell carcinoma (OSCC) (control group), Group 2 - patient with betel nut chewing habit with no history of OSF and OSCC, Group 3 - patient with betel nut chewing habit as a known case ISF and Group 4 - patient with betel nut chewing habit with a positive biopsy report for OSCC. Inclusion criteria were according to the study groups, patients with and without betel nut chewing habits; and with and without OSF and OSCC were included in the study. Also, patients between the ages of 18 and 45 years were included.

Exclusion criteria include a patient on drugs that cause liver toxicity, a patient with a history of liver toxicity, patient with any other co-morbid condition, patient who should not have regular smoking or alcohol drinking, and patient with history of Hepatitis B virus (HBV) infection, hepatocellular cancer, and liver transplant.

60 subjects were selected in total from the outpatient clinic of Oral Medicine and Radiology; that is 15 from each group. Informed consent was obtained by each subject before commencing the study. We designed a structured questionnaire to obtain information on age, sex, educational level, and practices of chewing betel nut. Chewing one or more BQs every day for at least a year was considered to be habitual. Detailed case history in view of liver function is taken by each patient with the help of a questionnaire (Table 1).

**Table 1:** Case history proforma

Date and Time -	Registration no.-
Name -	Age and Sex -
Religion -	Marital status -
Occupation -	Mobile no.-
Address-	
Chief complaint –	
History of presenting illness-	
Past medical history –	
Family history -	
Personal history –	
Habit history -	
General examination –	
Temperature –	Pulse rate-
Respiratory rate -	BP-
SpO_2_-	Pallor-
Icterus-	Cyanosis-
Clubbing-	Oedema-
Lymphadenopathy -	
Extraoral examination-	
Facial symmetry-	Lips-
TMJ-	Intraoral examination

Soft tissue examination-
Buccal mucosa
Labial mucosa-
Tongue-
Palate-
Floor of mouth-
OSF features – No. of bands- Burning sensation- Blanching of oral mucosa- Difficulty in swallowing- Inability to whistle /blow- Dry mouth- Mouth opening Grade -
Hard tissue examination-
Teeth present-
Teeth missing –
Wasting disorder-
Systemic examination – Liver examination
Inspection-
Palpation-
Percussion-
Auscultation-
Provisional diagnosis-
Investigation – Liver function test
Final diagnosis-
Patient sign - Doctor sign-

After taking the case history blood samples of each subject were collected in proper aseptic conditions with no harm to the subject. The collected samples were sent to the central laboratory of Acharya Vinoba Bhave Rural Hospital, Sawangi, Wardha, for the liver function test of each sample. After the collection of reports from the labs, the aspartate aminotransferase (AST) and alkaline phosphatase (ALP) levels were assessed, analyzed, and co-related with the case history of each subject and then the results will be derived.

Statistical method

All the results were calculated using Statistical Package for the Social Sciences (SPSS) version 25 (IBM Corp., Armonk, NY). Association between gender and liver palpable among the exposed group result using the chi-square test for finding the significance. Additionally, analyzed variance (ANOVA) to investigate whether there is a substantial age difference among the exposed groups, with a p-value threshold set at 0.05 for statistical significance; also delved into the mean levels of essential biomarkers, including AST, ALP, and protein, and bilirubin, among the exposed groups using ANOVA. Significance was determined at p≤0.05. Moreover, you are planning to evaluate the connections between the four study groups and the occurrence of liver palpability, which were correlated with the levels of AST and ALP enzymes, using a chi-square test of independence with a significance level of α=0.05. This comprehensive analysis aims to uncover significant associations and differences within the study data, with careful consideration of p-values and the chosen significance level for making informed conclusions.

## Results

A total of 60 subjects were included in the analysis, in which 15 subjects of each group (control group, patient with betel nut chewing habit with a history of OSF and OSCC, patient with betel nut chewing habit as a known case of OSF, patient with betel nut chewing habit and a known case of OSCC) were included. The sample included 45 male and 15 female subjects.

In this result, Table 1 shows that there is a high percentage of liver palpable seen in 2, 3, and 4 groups, which is a sign of liver toxicity. This shows that without disease in group 2 (patients with betel nut chewing habit without disease), there is 20% liver palpability seen; and with disease in groups 3 and 4 there is 26.7% and 33.3% liver palpability seen in the subjects which is significantly high.

**Table 2:** Liver palpability seen in four study groups

General parameters			Group				Total	Satistical Value	
			1.00	2.00	3.00	4.00		Chi.Sq.	P-value
Liver Palpable	MILD	Frequency	0	1	0	0	1	6.475	0.372
		%	0.0%	6.7%	0.0%	0.0%	1.7%		
	NO	Frequency	14	11	11	10	46		
		%	93.3%	73.3%	73.3%	66.7%	76.7%		
	YE	Frequency	1	3	4	5	13		
		%	6.7%	20.0%	26.7%	33.3%	21.7%		
Total		Frequency	15	15	15	15	60		
		%	100.0%	100.0%	100.0%	100.0%	100.0%		

Table 2 shows the AST level in all four study groups; the ANOVA test is for parametric data and here author shows frequency distribution and applies the chi-square test. The analysis result indicated a statistically significant difference between group averages for AST level was 3.437, with a significance value of 0.276, which was slightly more than 0.05. In Table 2, we can also appreciate that there is seen that out of 60 subjects, 13 subjects (21.7%) show an increase in AST level, and it is also appreciated that a greater number of affected subjects are seen from the second, third, and fourth groups.

**Table 3:** Aspartate aminotransferase levels seen in four study groups

			GROUPS				Total		
			1	2	3	4		Chi.Sq.	P-value
AST LEVEL	NO	Frequency	14	12	11	10	47	3.437	.276
		%	93.3%	80.0%	73.3%	66.7%	78.3%		
	YES	Frequency	1	3	4	5	13		
		%	6.7%	20.0%	26.7%	33.3%	21.7%		
Total		Frequency	15	15	15	15	60		
		%	100.0%	100.0%	100.0%	100.0%	100.0%		

Table 3 shows ALP levels in all four study groups, The ANOVA analysis result suggests a statistically significant difference between group averages for ALP level was 1.779, with a significance value of 0.628, which was slightly more than 0.05. In Table 3, we can also appreciate that there is seen that out of 60 subjects, seven subjects (11.7%) show an increase in ALP level, and it is also appreciated that a greater number of affected subjects are seen from the second and third groups.

**Table 4:** Alkaline phosphatase levels seen in four study groups

			GROUPS				Total		
			1	2	3	4		Chi.Sq.	P-value
ALP LEVEL	NO	Frequency	14	12	13	14	53	1.779	.628
		%	93.3%	80.0%	86.7%	93.3%	88.3%		
	YES	Frequency	1	3	2	1	7		
		%	6.7%	20.0%	13.3%	6.7%	11.7%		
Total		Frequency	15	15	15	15	60		
		%	100.0%	100.0%	100.0%	100.0%	100.0%		

The summary of frequency measurements revealed various distributions based on factors and associated categories. The chi-square test for independence was employed to determine if there was a link between the for-study groups and liver characteristics. The results revealed that the independent variables AST level, ALP level, and liver palpability were not statistically significant in any of the four research groups. Though the factors are not significant, it is noted that liver damage is present in all research groups, with betel nut chewing being more frequent in the group.

## Discussion

Betel nut and its derivatives are extensively utilized as a product for masticatory use in many communities and nations across the world. Over time, many additions were added to a simple betel nut mixture, resulting in the BQ [12]; which is vigorously chewed for a lengthy duration, thereby remaining in touch with the mucosa. The quid habit has an important social and cultural function in cultures across the Western Pacific, Southeast Asia, and Indian subcontinent [13]. Several studies show overwhelming evidence that areca nut is the primary etiological cause of OSF [8]. Betel chewing has been shown to alter parasympathetic, GABAergic, and sympathetic activities, as well as raise heart rate, blood pressure, perspiration, and body temperature. Furthermore, the electroencephalogram reveals broad cortical desynchronization, suggesting a state of excitement. Chewing betel raises plasma norepinephrine and epinephrine concentrations [14]. The hunger for BQ and its psychotropic effects, stress alleviation, and BQ's function in socializing are the most essential reasons for the inability to quit BQ chewing. Overcoming withdrawal symptoms and declining invites from others are the grounds for success [15].

The betel leaf, on the other hand, has significant quantities (15 m-1g fresh weight) of safrole, which is known to induce liver cancer in rats [7]. Hsiao et al. discovered that betel nut eating is an independent risk factor for liver cirrhosis [16].

In 2021, Shao et al. investigated the relationship between clinicopathologic factors of OSCC and betel nut chewing, and it was also revealed that ki-67 and p53 protein are little expressed in patients with OSCC who consume betel nuts, meaning that clinicopathologic parameters such as tumor growth, malignancy, differentiation, and prognosis are significantly improved.

The degree of differentiation is pretty excellent in OSCC patients who consume betel nuts [17]. Cirrhosis and hepatocellular carcinoma chances were increased in betel chewers who did not have hepatitis B/C infection [18]. Several studies have linked betel chewing to an increased risk of a variety of health issues, including liver cirrhosis and hepatocellular cancer [19]. ALT is the blood enzyme seen in clinical chemistry panels that is most suggestive of hepatocellular necrosis. Although less specific, AST is also employed. Elevated bilirubin in serum also indicates cholestasis and decreased protein levels indicate hepatotoxicity [20]. Betel nut chewing leads to OSF, OSCC, and liver cancer. And levels of AST and ALT are clinical chemistry panels that are most suggestive of hepatocellular necrosis.

This study uses a rigorous epidemiological technique to show that routinely chewing betel nuts is a risk factor for liver damage. This research will aid in establishing a link between the practice of chewing betel nuts and the occurrence of OSF, OSCC, and liver damage. Regularly chewing betel nuts appears to be a separate risk factor for liver damage and OSF.

OSF was characterized by the presence of palpable fibrous bands in the oral mucosa leading to limited mouth opening (i.e., trismus). Intolerance to spicy food and oral ulceration were other common features of OSF [21]. By the statistical analysis, we can appreciate that in PSF patients the ALP levels are raised, suggestive of liver toxicity; this suggests that we can diagnose liver toxicity at an early stage by running the liver function test for the OSF patients.

Adverse immune-related liver events can complicate immunotherapy for metastatic cancer. The diagnosis and assessment of the severity of liver impairment in patients with immune-mediated hepatitis who are undergoing immunotherapy for metastatic cancer are aided by liver biopsy [22]. The results also show that there is liver toxicity seen in a group of patients with oral cancer.

Also, we can appreciate that liver toxicity and OSF/OSCC can be diagnosed at the same time in patients with the betel nut chewing habit. Therefore, by suspecting liver toxicity in betel nut chewers we can decrease the prevalence of severe liver toxicity in the patients and also treat the disease in an early stage, and also stop the further progration of the disease. The limitation of this study is that it does not include all types of cancers, the study groups have age limitations; also, and the number of subjects is very low.

## Conclusions

Hence, this assessment of liver function with case history in each subject will aid in providing an improvised and prioritized method for the early diagnosis of liver misfunctioning in the patient with OSF or OSCC due to a common etiological factor, that is betel nut. Also, we can see that in patients who eat betel nuts, liver damage and OSF/OSCC can be identified at the same time. Hence, the treatment for the patients most in need and are likely to benefit from the early diagnosis of the pathology.
